# Fluoroscopy usage in contemporary interventional electrophysiology: Insights from a European registry

**DOI:** 10.1002/clc.23411

**Published:** 2020-11-21

**Authors:** Jedrzej Kosiuk, Lucas Fiedler, Sabine Ernst, David Duncker, Nikola Pavlović, Silvia Guarguagli, Clara Stegmann, Dawid Miskowiec, Rodrigue Garcia, Vincenzo Russo, Andriy Yakushev, Nándor Szegedi, Tom De Potter

**Affiliations:** ^1^ Rhythmology Department Helios Clinic Koethen Koethen Germany; ^2^ Department of Internal Medicine II General Hospital Wiener Neustadt Wiener Neustadt Austria; ^3^ Royal Brompton Hospital London UK; ^4^ Rhythmology and Electrophysiology, Department of Cardiology and Angiology Hannover Medical School Hannover Germany; ^5^ Department of Cardiology Univeristy Hospital Center Sestre Milosrdnice Zagreb Croatia; ^6^ Department of Electrophysiology Heart Center Leipzig Leipzig Germany; ^7^ Department of Cardiology Medical University of Lodz Lodz Poland; ^8^ Rhythmology Department CHU de Poitiers Poitiers France; ^9^ Department of Translational Medical Sciences University of Campania "Luigi Vanvitelli"—Monaldi Hospital Naples Italy; ^10^ Amosov National Institute of Cardiovascular Surgery Kyiv Ukraine; ^11^ Heart and Vascular Center Semmelweis University Budapest Hungary; ^12^ Cardiovascular Center, OLV Hospital Aalst Belgium

**Keywords:** electrophysiology, fluoroscopy, radiation, x‐ray, zero‐fluoroscopy

## Abstract

**Background:**

Fluoroscopy has been an essential part of every electrophysiological procedure since its inception. However, till now no clear standards regarding acceptable x‐ray exposure nor recommendation how to achieve them have been proposed.

**Hypothesis:**

Current norms and quality markers required for optimal clinical routine can be identified.

**Methods:**

Centers participating in this Europe‐wide multicenter, prospective registry were requested to provide characteristics of the center, operators, technical equipment as well as procedural settings of consecutive cases.

**Results:**

Twenty‐five centers (72% university clinics, with a mean volume of 526 ± 348 procedures yearly) from 14 European countries provided data on 1788 cases [9% diagnostic procedures (DP), 38% atrial fibrillation (AF) ablations, 44% other supraventricular (SVT) ablations, and 9% ventricular ablations (VT)] conducted by 95 operators (89% male, 41 ± 7 years old).

Mean dose area product (DAP) and time was 304 ± 608 cGy*cm^2^, 3.6 ± 4.8 minutes, 1937 ± 608 cGy*cm^2^, 15.3 ± 15.5 minutes, 805 ± 1442 cGy*cm^2^, 10.6 ± 10.7 minutes, and 1277 ± 1931 cGy*cm^2^, 10.4 ± 12.3 minutes for DP, AF, SVT, and VT ablations, respectively. Seven percent of all procedures were conducted without any use of fluoroscopy.

Procedures in the lower quartile of DAP were performed more frequently by female operators (OR 1.707, 95%CI 1.257‐2.318, *P* = .001), in higher‐volume center (OR 1.001 per one additional procedure, 95%CI 1.000‐1.001, *P* = .002), with the use of 3D‐mapping system (OR 2.622, 95%CI 2.053‐3.347, *P* < .001) and monoplane x‐ray system (OR 2.945, 95%CI 2.149‐4.037, *P* < .001).

**Conclusion:**

Exposure to ionizing radiation varies widely in daily practice for all procedure. Significant opportunities for harmonization of exposure toward the lower range has been identified.

## 1 INTRODUCTION

Interventional treatment of arrhythmias with catheter ablation is still commonly performed under fluoroscopic guidance resulting in exposure of patients and personnel to ionizing radiation. Exposure to ionizing radiation may be harmful both for personnel and for patients, with inherent risk of neoplasms due to long‐term exposure representing the biggest concern.^1^ Electrophysiology procedures were initially performed solely with the use of fluoroscopy but the development of novel mapping systems has led to a dramatic decrease or even a complete abandonment of fluoroscopy during the last decade.^2^ The U.S. Nuclear Regulatory Commission recommends making every effort to keep exposure to ionizing radiation as low as reasonably achievable (ALARA), a statement that is accepted and endorsed by all the major societies of physicians working with ionizing radiation.^1^, ^2^, ^3^ The purpose of “Go for Zero Fluoroscopy” project is to assess current routine and practice with the use of fluoroscopy in electrophysiology centers and to identify factors associated with low radiation dose during procedures.

## 2 METHODS

### 2.1 Primary objective

The study aimed to describe the real‐life, contemporary use of fluoroscopy in interventional electrophysiology across European countries. The main goal of this analysis was to identify factors associated with the low dose of radiation defined as procedures within the lower quartile of DAP.

### 2.2 Study design and setting

The Go for Zero Fluoroscopy project is a prospective, international, observational registry of consecutive patients undergoing any type of interventional electrophysiological procedure. Design, oversight, and logistics was conducted under the “Go for Zero Fluoroscopy Project” of the European Hearth Rhythm Association's Young EP committee (EHRA YEP). The study was conducted in 25 centers from 14 European countries (full list provided in the Appendix 1). Local principal investigators obtained approval by the local Institutional Review Board, depending on regulations in each country.

### 2.3 Study participants

Centers were asked to provide anonymized data regarding the procedural setting of maximum 20 consecutive electrophysiological interventions conducted by maximum five different operators. This limit was introduced in order to prevent statistical bias resulting from data overflow from high‐volume centers or operators. Furthermore, precise description of the operator characteristics as well center characteristics was recorded.

### 2.4 Data collection

All centers were asked to complete a one‐time site questionnaire consisting of three parts (a) description of the center, (b) operators characteristics (c) procedural setting (details of the questionnaire are provided in Appendix 2).

Data were collected using a web‐based system with automatic validation algorithm.

### 2.5 Statistical analysis

Continuous variables are presented as mean ± one SD, and categorical variables as frequencies. Continuous variables were compared using the Student's *t*‐test, or nonparametric tests in case of non‐normal distribution (tested with Kolmogorov‐Smirnov test). Categorical variables were compared using the chi‐square test. To calculate odds ratio (OR) a logistic regression model was used. A *P*‐value of < .05 was considered statistically significant. Analysis was performed with SPSS v 20.0 (SPSS Inc., Chicago, Illinois).

## 3 RESULTS

### 3.1 Characteristics of participating centers and operators

Out of the 25 participating centers 18 were university hospitals (72%) with an annual volume of 526 ± 349 procedures, followed by public hospitals 5 (20%) and tertiary nonacademic institutions 2 (8%) with a mode of 5 [3–6] electrophysiologist working in the department. The study was conducted across 14 European countries: three centers (12%) in France, Germany, Italy, Poland, two centers (8%) in Austria, Croatia, Spain, and one center (4%) in Belgium, Hungary, Portugal, Romania, Slovenia, Ukraine, and United Kingdom.

Majority of the 95 participating operators were male [n = 81 (85%)] with mean age of 41 ± 8 years. Level of experience varied strongly among the population as the IQR for completion of the training spanned between 2 and 10 years with median of 5 years. Most of the operators were at the mid‐career (>5 years) level [41 (43%)], followed by early‐career (<5 years) level [31 (33%)] and mentor (>15 years) level [23 (24%)]. The number of conducted procedures per single operator was as follows: 1 to 9 procedures per month in 31% of the cases, 10 to 19 in 25%, 20 to 40 in 22%, and >40 in 18%. Only 18 operators (19%) also conducted coronary interventions on a regular basis, while the majority were also performing device implantations [70 (74%)].

Detailed characteristics of the centers and operators are summarized in Tables 1 and 2.

**TABLE 1:** Center characteristics (n = 25)

Type of the center	
Public hospital	5 (20%)
Tertiary nonacademic	2 (8%)
University hospital	18 (72%)
Country	
Austria	2 (8%)
Belgium	1 (4%)
Croatia	2 8 (%)
France	3 (12%)
Germany	3 (12%)
Hungary	1 (4%)
Italy	3 (12%)
Poland	3 (12%)
Portugal	1 (4%)
Romania	1 (4%)
Slovenia	1 (4%)
Spain	2 (8%)
Ukraine	1 (4%)
United Kingdom	1 (4%)
Number of procedures per year (n ± SD)	526 ± 349
Number of electrophysiologists, mode [IQR]	5 [3‐6]

**TABLE 2:** Operator characteristics (n = 95)

Age, (years)	41 ± 8
Male, n (%)	81 (85)
Years out of training, (years)	5 [2‐10]
Numbers of procedure per month, n (%)	
1‐9	29 (31)
10‐19	24 (25)
20‐40	21 (22)
>40	17 (18)
Level of experience in EP, n (%)	
Early‐career (<5 years)	31 (33)
Mid‐career (>5 years)	41 (43)
Mentor (>15 years)	23 (24)
Also conducting coronary interventions, n (%)	18 (19)
Also conducting device interventions, n (%)	70 (74)

### 3.2 Procedural settings

The most frequently conducted procedure in the current study was ablation of supraventricular tachycardia [794 (44%)], followed by atrial fibrillation ablation [683 (38%)], ventricular tachycardia [157 (9%)] and diagnostic procedure [154 (9%)]. The mean procedural duration was 110 ± 65 minutes. During the majority of the procedures [966 (54%)], some type of 3D mapping system was used. Contact force technology was widely utilized [747 (42%)] while only in 141 cases (8%) a dedicated multipolar mapping catheter was implemented. Remote navigation was used in seven cases (1%).

In 275 cases (15%), fusion with an additional image modality was employed. One hundred and ninety‐six cases (11%) were conducted under general anesthesia.

Detailed case characteristic is summarized in Table 3.

**TABLE 3:** Procedure‐related data (n = 1788)

Type of EP procedure	n (%)
Atrial fibrillation ablation	683 (38)
Pulmonary vein isolation (RF)	392 (22)
Pulmonary vein isolation + additional lesions (RF)	122 (7)
Pulmonary vein isolation (single shot device)	126 (7)
Left sided AT	42 (2)
Supraventricular tachycardia, n (%)	794 (44)
AV node	66 (4)
AVNRT	267 (15)
Typical flutter	301 (17)
Right sided AT	38 (2)
Left sided AP	64 (4)
Right sided AP	41 (2)
Scar‐related SVT	17 (1)
Ventricular tachycardia, n (%)	157 (9)
Scar‐related VT	66 (4)
PVC	91 (5)
Diagnostic procedure	154 (9)
Procedure duration (min ± SD)	110 ± 65
Fluoroscopy time (min ± SD)	12 ± 13
Dose area product (cGy*cm2 ± SD)	1236 ± 2295
Procedures without use of fluoroscopy, n (%)	125 (7%)
Type of 3D mapping system, n (%)	
None	822 (46)
Carto	549 (31)
NavX	293 (16)
Rhythmia	35 (2)
Other	89 (5)
Contact force, n (%)	747 (42)
Multipolar mapping catheter, n (%)	141 (8)
Remote navigation, n (%)	7 (1)
Image fusion, n (%)	275 (15)
General anesthesia, n (%)	195 (11)
Presence of X‐ray technician, n (%)	814 (46)
Type of X‐ray installation, n (%)	
Biplane	326 (18)
Monoplane	1366 (76)
X‐ray C‐arm	96 (5)

### 3.3 Use of fluoroscopy

The mean fluoroscopy time was 12 ± 13 minutes during which a mean DAP of 1236 ± 2295 cGy*cm^2^ was delivered. Both values varied strongly between different types of procedures: 304 ± 608 cGy*cm^2^, 3.6 ± 4.8 minutes for dose area product (DP) and 1937 ± 608 cGy*cm^2^, 15.3 ± 15.5 minutes, 805 ± 1442 cGy*cm^2^, 10.6 ± 10.7 minutes, 1277 ± 1931 cGy*cm^2^, 10.4 ± 12.3 minutes for AF, SVT, and VT ablation, respectively. Among 1788 cases, 117 procedures (7%) were conducted without any use of fluoroscopy: 12 DP, 87 SVT, 7 AF, and 11 VT. In 814 cases (46%), a dedicated x‐ray technician was present during the complete procedure. In 1366 (76%) cases, a monoplane X‐ray installation was used.

The correlation between applied dose of fluoroscopy and time of exposure was poor, although statistically significant (*R* ^2^ = .37, *P* < .001).

**FIGURE 1:**
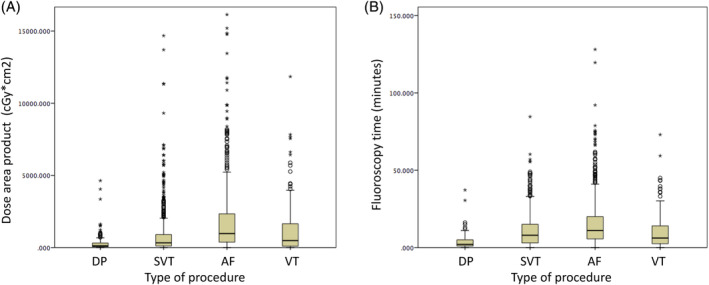
Mean fluoroscopy use across different procedure types: A, DAP and B, fluoroscopy time

The use of fluoroscopy is presented in Figure 1.

### 3.4 Factors associated with reduced use of fluoroscopy

Procedures in lower quartile of DAP were characterized by values <52 cGy*cm^2^ for DP, < 110 cGy*cm^2^ for SVT cases, <325 cGy*cm^2^ for AF ablation and <972 cGy*cm^2^ for VT procedures.

In univariate analysis, female gender was significantly associated with a reduced dose of fluoroscopy (OR 1.599, 95%CI 1.202‐2.127, *P* = .001). Furthermore, the number of procedures conducted by the operator on monthly basis (OR 1.285, 95%CI 1.017‐1.623, *P* = .035 for >10 cases) as well the level of experience (OR 1.219, 95%CI 1.019‐1.464, *P* = .031 for >5 years) were also associated with lower dose of fluoroscopy.

Use of a 3D‐mapping system (OR 2.775, 95% CI 2.199‐3.503, *P* < .001) as well as monoplane X‐ray equipment (OR 2.310, 95% CI 1.718‐3.105, *P* < .001) were significantly associated with reduced dose of fluoroscopy.

Finally, in centers with higher volume, procedures were more frequently conducted within the lower DAP quartile (522 ± 355 vs 606 ± 395, *P* < .001).

In the multivariate analysis only female gender (OR 1.707, 95% CI 1.257‐2.318, *P* = .001) characterized operators conducting lower exposure procedures. Procedural setting including use of 3D‐mapping system (OR 2.622, 95% CI 2.053‐3.347, *P* < .001) and monoplane X‐ray equipment (OR 2.945, 95% CI 2.149‐4.037, *P* < .001) remained also significantly associated with lower dose of fluoroscopy. Furthermore, a significant correlation between number of procedures performed in a center and a lower exposure has been verified (OR 1.001 per one additional procedure, 95% CI 1.000‐1.001, *P* = .002).

**FIGURE 2:**
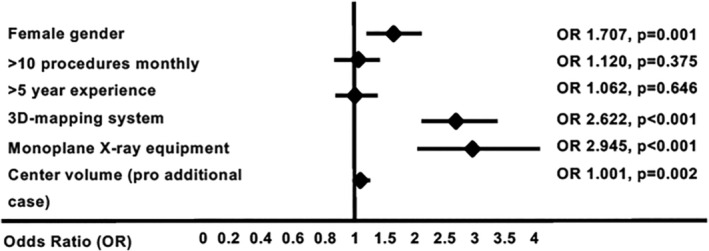
Result of multivariate analysis

The results of multivariate analysis are presented in Figure 2.

## 4 DISCUSSION

### 4.1 Main findings

The main goal of this study was to evaluate use of fluoroscopy in contemporary EP centers throughout Europe. An important finding of this prospective, international registry is that female gender, the use of a 3D‐mapping system, monoplane X‐ray equipment and procedures performed in a high‐volume center are predictors of lower fluoroscopy exposure during invasive electrophysiological procedures.

### 4.2 Importance of fluoroscopy dose reduction

Electrophysiology procedures are traditionally performed under fluoroscopic guidance and are associated with ionizing radiation exposure that may result a non‐negligible health risk for both patients and laboratory staff.^4^, ^5^ Radiological exposure is a hot topic nowadays as cardiology is responsible for about 40% of the entire exposure from all medical sources, as a consequence of widespread availability of X‐ray‐based imaging techniques.^6^, ^7^, ^8^, ^9^ Cardiac use of fluoroscopy almost never reaches the threshold for deterministic radiation injury, but it gives an additional lifetime risk of fatal and nonfatal cancers thus the amount of used radiation should be as low as reasonably achievable.^10^ A cornerstone of enhanced radiation safety is optimization, that is, reducing as much as possible the use of X‐rays for a given technique.^6^, ^7^, ^8^, ^9^, ^11^

### 4.3 Different units of measurement

As the awareness of the relevance of fluoroscopy gets higher and higher there is a need for a more precise description of radiation exposure. Even these days many authors use the description of “fluoroscopy time” as a measure of radiation related to ablation procedures. However, this may be misleading as there are many other factors that influence radiation exposure such as frame rate, radiation dose per pulse, collimation—each of which will determine effective dose to a far greater degree than exposure time.^12^, ^13^ In line with this statement, our registry also showed only a weak correlation between fluoroscopy time and dose. Because dose area product is readily available from all imaging system vendors, and because it provides an exposure dose that linearly correlates with true estimated effective biological dose, it is probably a far better parameter for comparing radiation exposure in this and other datasets.^14^

### 4.4 Dose of fluoroscopy—low, near zero, and zero

The goal of reducing exposure to ionizing radiation as much as possible is well accepted. Considering the stochastic effects of radiation it is not likely that there will ever be a recommendation for a radiation dose that is considered “safe,” as very low doses may still be harmful. Thus, current best practice is to get close to (“near zero”) or ideally achieve zero fluoroscopy. After numerous single‐center experience papers and feasibility studies the first prospective randomized trial (NO‐PARTY trial) showed that a minimally fluoroscopic approach significantly reduces radiation exposure during electrophysiology procedures while it does not compromise procedure time and does not affect safety and efficacy.^15^

Obviously, “near zero” and zero fluoroscopy involves an expense both in terms of time (learning) as well as equipment, as additional imaging modalities such as electroanatomical mapping systems or intracardiac echocardiography (ICE) are typically used in these cases.

Of note, a zero (or near zero) fluoro approach requires individual training but the learning curve can be relatively short especially if the physician is familiar with the use of electroanatomical mapping systems and/or ICE. Nowadays all types of procedures can be performed with a (near) zero fluoroscopy approach, even complex ablations such as pulmonary vein isolation—although some form of imaging such as ICE or transesophageal echo might be necessary to perform specific procedural steps such as transseptal puncture.^16^

### 4.5 European overview through our registry

The present registry provides a diverse European overview as 25 EP laboratories from 14 countries across Europe were involved. High and low volume centers are also represented, although the majority of centers are university hospitals. A limit of five operators per center and 20 consecutive cases per operator was set to avoid over‐representation of high volume centers and high volume operators. In this registry, we observed that fluoroscopy doses are in a lower range as compared to previous reports which may indicate a favorable trend toward a better clinical practice in terms of reducing the use of ionizing radiation as much as possible.^17^ Moreover, in our registry 7% of all procedures were performed without the use of any fluoroscopy. Multivariate analysis showed high volume centers and use of monoplane systems to be independent predictors of lower DAP values which is likely to be attributable to available expertise and availability of mapping systems in those centers. Of particular note is that female gender is the only independent predictor of low exposure on the operator level. The authors therefore speculate that a personal motivation for radiation reduction seems to be a significant driver in achieving low fluoroscopy exposure.^18^

### 4.6 Limitations

The main limitation of our dataset is its observational nature. However, real‐life registries of consecutive patients at the level of individual operators have the potential to provide insights into contemporary practice across different regions with different practices, regulatory requirements, and resources. All data were prospectively collected using the same method in all participating centers, but some datapoints were incomplete for a minority of procedures.

## 5 CONCLUSIONS

This real‐life registry of fluoroscopy usage across Europe shows that contemporary practice and use of modern mapping technology results in average exposure doses below previously reported values. Furthermore, gender and thus probably personal motivation is independently associated with lower radiation exposure.

## CONFLICT OF INTEREST

The authors declare no potential conflict of interests.

## Supporting information


**Appendix** **S1**: Supporting Information

**Appendix** **S2**: Supporting Information

## References

[1] Klein LW , Miller DL , Balter S , et al. Occupational health hazards in the interventional laboratory: time for a safer environment. Heart Rhythm. 2009;6:439‐444. 19201659 10.1016/j.hrthm.2009.01.030

[2] Knecht S , Sticherling C , Reichlin T , et al. Effective reduction of fluoroscopy duration by using an advanced electroanatomic‐mapping system and a standardized procedural protocol for ablation of atrial fibrillation: the unleaded study. Europace. 2015;17:1694‐1699. 25995391 10.1093/europace/euv006

[3] Santoro A , Di Clemente F , Baiocchi C , et al. From near‐zero to zero fluoroscopy catheter ablation procedures. J Cardiovasc Electrophysiol. 2019;30:2397‐2404. 10.1111/jce.14121. 31424119

[4] Picano E , Vano E . The radiation issue in cardiology: the time for action is now. Cardiovasc Ultrasound. 2011;9:35. 22104562 10.1186/1476-7120-9-35 PMC3256101

[5] Chen J , Einstein AJ , Fazel R , et al. Cumulative exposure to ionizing radiation from diagnostic and therapeutic cardiac imaging procedures: a population‐based analysis. J Am Coll Cardiol. 2010;56:702‐711. 20619569 10.1016/j.jacc.2010.05.014 PMC2952402

[6] Estner HL , Grazia Bongiorni M , Chen J , et al. Use of fluoroscopy in clinical electrophysiology in Europe: results of the European Heart Rhythm Association Survey. Europace. 2015;17:1149‐1152. 26116687 10.1093/europace/euv223

[7] Picano E , Vano E , Rehani MM , et al. The appropriate and justified use of medical radiation in cardiovascular imaging: a position document of the ESC Associations of Cardiovascular Imaging, Percutaneous Cardiovascular Interventions and Electrophysiology. Eur Heart J. 2014;35:665‐672. 24401558 10.1093/eurheartj/eht394

[8] Fazel R , Gerber TC , Balter S , et al. Approaches to enhancing radiation safety in cardiovascular imaging: a scientific statement from the American Heart Association. Circulation. 2014;130:1730‐1748. 25366837 10.1161/CIR.0000000000000048

[9] Natarajan MK , Paul N , Mercuri M , et al. Canadian cardiovascular society position statement on radiation exposure from cardiac imaging and interventional procedures. Can J Cardiol. 2013;29:1361‐1368. 24035289 10.1016/j.cjca.2013.06.002

[10] Linet MS , Kim KP , Miller DL , Kleinerman RA , Simon SL , Berrington de Gonzalez A . Historical review of occupational exposures and cancer risks in medical radiation workers. Radiat Res. 2010;174:793‐808. 21128805 10.1667/RR2014.1 PMC4098897

[11] Szegedi N , Zima E , Clemens M , et al. Radiofrequency ablation of focal atrial tachycardia: benefit of electroanatomical mapping over conventional mapping. Acta Physiol Hung. 2015;102:252‐262. 26551741 10.1556/036.102.2015.3.3

[12] Thibault B , Macle L , Mondesert B , et al. Reducing radiation exposure during procedures performed in the electrophysiology laboratory. J Cardiovasc Electrophysiol. 2018;29(2):308‐315. 29064134 10.1111/jce.13373

[13] Rubesch‐Kutemeyer V , Fischbach T , Guckel D , et al. Long‐term development of radiation exposure, fluoroscopy time and contrast media use in daily routine in cryoballoon ablations after implementation of intracardiac echocardiography and other radioprotective measures: experiences from a large single‐centre cohort. J Interv Card Electrophysiol. 2019;4(3):10840‐10848. 10.1007/s10840-019-00564-5 31168672

[14] Heidbuchel H , Wittkampf FH , Vano E , et al. Practical ways to reduce radiation dose for patients and staff during device implantations and electrophysiological procedures. Europace. 2014;16(7):946‐964. 24792380 10.1093/europace/eut409

[15] Casella M , Dello Russo A , Pelargonio G , et al. Near zerO fluoroscopic exPosure during catheter ablAtion of supRavenTricular arrhYthmias: the NO‐PARTY multicentre randomized trial. Europace. 2016;18(10):1565‐1572. 26559916 10.1093/europace/euv344 PMC5072134

[16] Bulava A , Hanis J , Eisenberger M . Catheter ablation of atrial fibrillation using zero‐fluoroscopy technique: a randomized trial. Pacing Clin Electrophysiol. 2015;38(7):797‐806. 25790320 10.1111/pace.12634

[17] Casella M , Dello Russo A , Russo E , et al. X‐ray exposure in cardiac electrophysiology: a retrospective analysis in 8150 patients over 7 years of activity in a modern, large‐volume laboratory. J Am Heart Assoc. 2018;7(11):e008233. 29789334 10.1161/JAHA.117.008233 PMC6015357

[18] Sarkozy A , De Potter T , Heidbuchel H , et al. Occupational radiation exposure in the electrophysiology laboratory with a focus on personnel with reproductive potential and during pregnancy: a European heart rhythm association (EHRA) consensus document endorsed by the Heart Rhythm Society (HRS). Europace. 2017;19(12):1909‐1922. 29126278 10.1093/europace/eux252

