# Supplementary material for: Fluoroscopy usage in contemporary interventional electrophysiology: Insights from a European registry
Source: Clin Cardiol. 2020 Nov 21;44(1):36–42. doi: 10.1002/clc.23411 (PMC7803367; doi:10.1002/clc.23411)
Supplement: Supplementary file 1 — Appendix S1: Supporting Information [file CLC-44-36-s001.docx]

Appendix 1

**List of participating centers and local PIs (in random order):**

1 Rhythmology Department, Helios Klinikum Köthen, Köthen, Germany, Jedrzej Kosiuk

2 Department of Internal Medicine II, General Hospital Wiener Neustadt, Wiener Neustadt, Austria, Lucas Fiedler

3 Rhythmology and Electrophysiology, Department of Cardiology and Angiology, Hannover Medical School, Carl-Neuberg-Str. 1, D-30625 Hannover, Germany, David Duncker

4 Department of Cardiology, Univeristy Hospital Center Sestre milosrdnice, Zagreb, Croatia, Nikola Pavlović

5 Royal Brompton Hospital, London, UK, Silvia Guarguagli

6 Department of Electrophysiology, Heart Center Leipzig, Leipzig, Germany, Clara Stegmann

7 Department of Cardiology, Medical University of Lodz, Poland, Dawid Miskowiec

8 Rhythmology Department, CHU de Poitiers, 86021, Poitiers, France, Rodrigue Garcia

9 Chair of Cardiology, Department of Translational Medical Sciences, University of Campania "Luigi Vanvitelli" - Monaldi Hospital, Naples, Italy, Vincenzo Russo

10 Amosov National institute of cardiovascular surgery, Kyiv, Ukraine, Andriy Yakushev

11 Heart and Vascular Center, Semmelweis University, Budapest, Hungary, Nándor Szegedi

12 Cardiovascular Center, OLV Hospital, Aalst, Belgium, Tom De Potter

13 University Hospital Center Rijeka, Rijeka, Croatia, Sandro Brusich

14 1st Chair and Department of Cardiology, Warsaw, Poland, Monika Gawałko

15 Silesian Center for Heart Diseases, Zabrze, Poland, Krzysztof Myrda

16 Hospital Universitario Bellvitge , Barcelona, Spain, Zoraida Moreno Weidmann

17 Kepler University Hospital, Department of Cardiology, Linz, Austria, Hermann Blessberger

18 Hospital Sta Cruz, Lisbon, Portugal, Francisco Costa

19 San Donato Hospital, Arezzo, Italy, Martina Nesti

20 Virgen de las Nieves Universitary Hospital, Grenada, Spain, Sanches Millan

21 University Medical Center Ljubljana, Ljubljana, Slovenia, Bor Antolic

22 Pitie salpetriere, Paris, France, Estelle Gandjbakhch

23 Clinic Fürth, Fuerth, Germany, Laura Vitali-Serdoz

24 European Georges Pompidou Hospital, Paris, France, Victor Waldmann

25 Emergency Clinical Hospital Bucharest, Bucharest, Romania, Stefan Bogdan
